# A Systematic and Narrative Review of Acupuncture Point Application Therapies in the Treatment of Allergic Rhinitis and Asthma during Dog Days

**DOI:** 10.1155/2015/846851

**Published:** 2015-10-12

**Authors:** Cai-Yu-Zhu Wen, Ya-Fei Liu, Li Zhou, Hong-Xing Zhang, Sheng-Hao Tu

**Affiliations:** ^1^Hubei University of Chinese Medicine, No. 1 Huangjiahu West Road, Wuhan, Hubei 430065, China; ^2^Department of Nephrology, The First Affiliated Hospital of Zhengzhou University, 1 Jianshe East Road, Zhengzhou, Henan 450052, China; ^3^Department of Acupuncture and Moxibustion, Wuhan Integrated TCM and Western Medicine Hospital, Hubei University of Chinese Medicine, No. 215 Zhongshan Avenue, Wuhan, Hubei 430022, China; ^4^Institute of Integrated Traditional Chinese and Western Medicine, Tongji Hospital, Tongji Medical College, Huazhong University of Science and Technology, 1095 Jiefang Avenue, Wuhan, Hubei 430030, China

## Abstract

Acupuncture point application therapies, including San-Fu-Tie and San-Fu-Jiu, have been widely employed to treat diseases with attacks in winter during dog days in China. The therapies combine Chinese herbal medicine and acupuncture points with the nature. However, the previous studies were reported to be unsystematic and incomplete. To develop a more comprehensive understanding of the effects of acupuncture point application therapies on allergic rhinitis and asthma, a systematic review of the literature up to 2015 was conducted. After filtering, eighteen randomized controlled trials (RCTs) involving 1,785 subjects were included. This systematic and narrative review shows that acupuncture point application therapies have been extensively applied in the treatment of allergic rhinitis and asthma with advantages of favorable treatment effect, convenient operation, receiving patients' good acceptability and compliance, and few side effects. Meanwhile, the study elaborated the operating process of San-Fu-Tie and San-Fu-Jiu in detail. The review may provide a reference for clinical application in future. However, the efficacy, safety, and mechanisms of San-Fu-Tie and San-Fu-Jiu in treating the above diseases need to be validated by more well-designed and fully powered RCTs in a larger population of patients.

## 1. Introduction

Acupuncture point application therapies, combining Chinese herbal medicine and acupuncture points during dog days, have been extensively applied for a long time in the treatment of allergic rhinitis (AR) and asthma [1]. Dog days are usually the three ten-day periods of the hottest season. They are divided into “1st dog day,” “2nd dog day,” and “3rd dog day.” According to the lunar calendar, the period of adjacent Geng day is ten days. The 3rd Geng day after the summer solstice is “1st dog day.” The 4th Geng day after the summer solstice and the 1st Geng day after the beginning of autumn are “2nd dog day” and “3rd dog day,” respectively.

**Figure 1:**
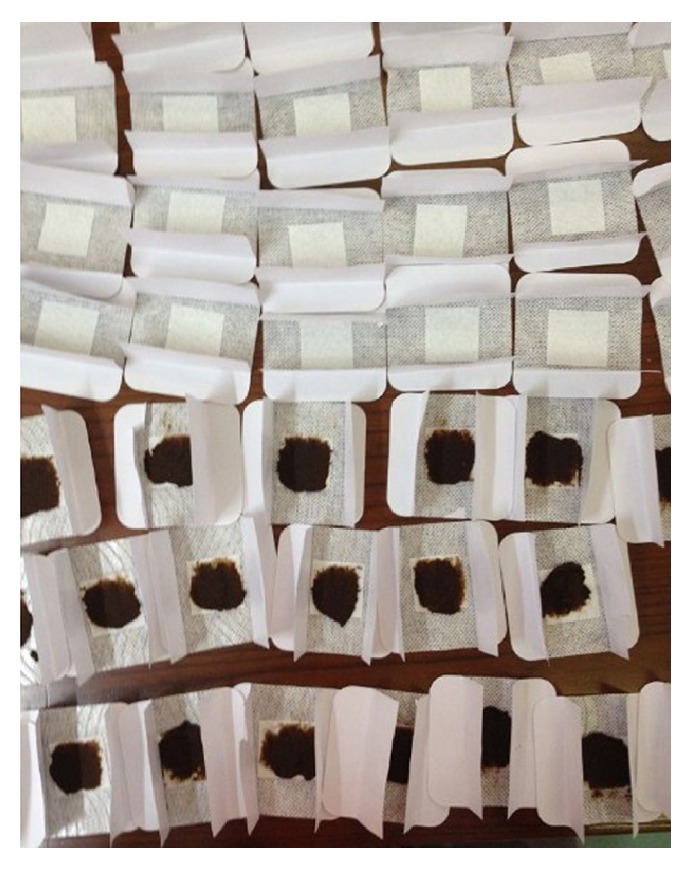
Chinese herb medicine pastes for San-Fu-Tie.

The therapies are predominantly comprised of San-Fu-Tie and San-Fu-Jiu. The former applies special Chinese herbal medicine paste to the acupuncture points [2]. The basic herbal prescription of San-Fu-Tie is usually composed of Bai Jie Zi (*Semen Sinapis Albae*), Xi Xin (*Herba Asari*), Gan Sui (*Radix Kansui*), and Yan Hu Suo (*Rhizowa Corydalis*) [1]. Based on the theory of syndrome differentiation of traditional Chinese medicine (TCM), other herbal medicines of similar effects are allowed to be applied according to different diseases. The herbal medicine is processed as follows: ground into powder, mixed with ginger juice or honey, divided into small cubes of 3–5 g each, and laid on the applications (Figure 1) [3–5]. The latter is the combination of ginger-separated moxibustion and acupoint application [6]. However, there is no clear distinction between San-Fu-Tie and San-Fu-Jiu in some hospitals, which is universally called San-Fu-Tie San-Fu-Jiu. On the contrary, they are considered different in some other hospitals. In this review, we consider that they are two different therapies.

**Figure 2:**
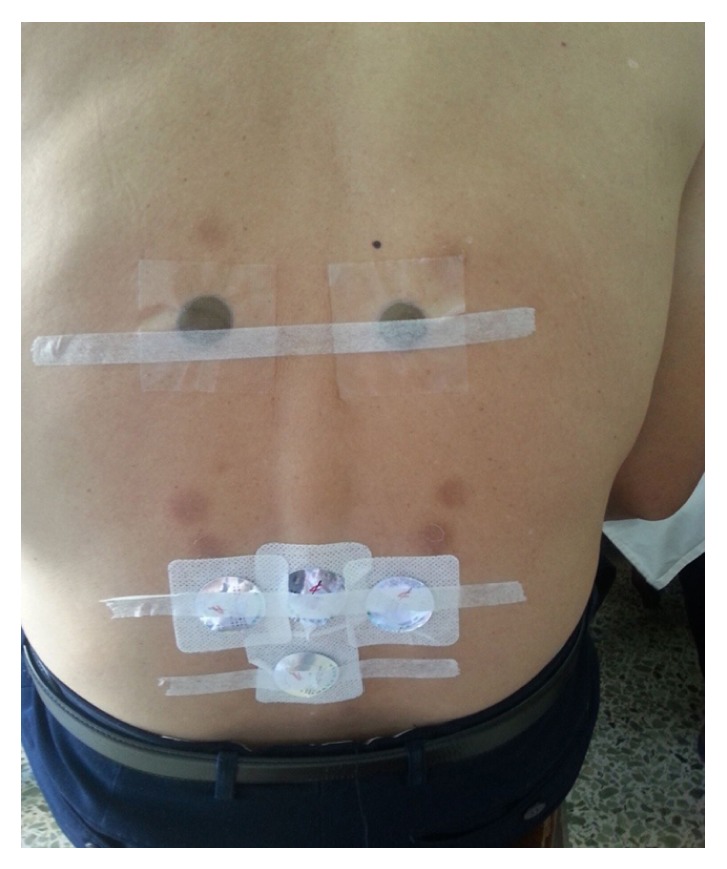
San-Fu-Tie was applied on Shenshu (BL23), Yaoyangguan (GV3), Mingmen (GV4), and Pishu (BL20).

**Figure 3:**
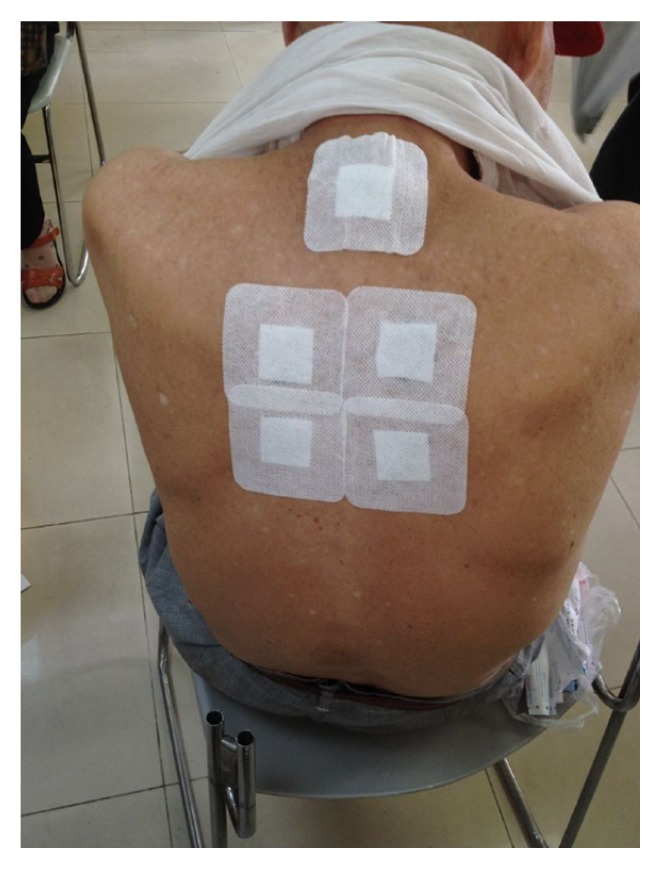
San-Fu-Tie was applied on Dazhui (GV14), Fengmen (BL12), and Feishu (BL13).

**Figure 4:**
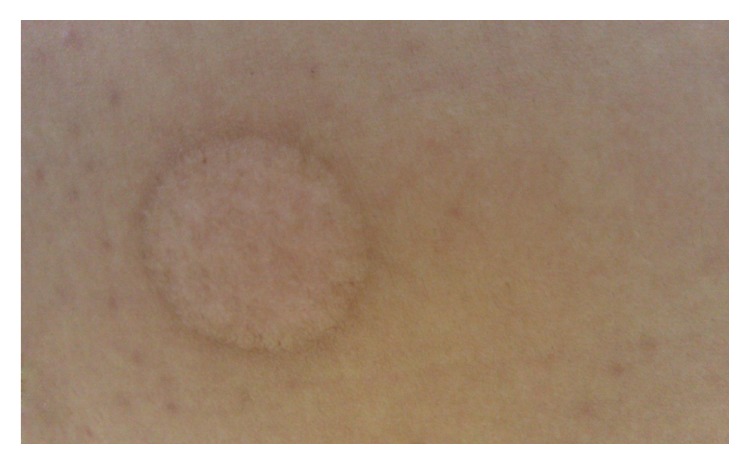
The left scar after San-Fu-Tie or San-Fu-Jiu.

The San-Fu-Tie is operated conforming to the following steps: firstly, the acupoints are selected according to different diseases; secondly, the prepared herbal medicine is placed onto the selected acupoints; thirdly, in order to prevent paste falling off, medical adhesive tapes are needed to reinforce the applications (Figures 2 and 3). Adults are always pasted for 4–6 hours in principle. Considering the delicate skin of children, they are often pasted for less than 2 hours to avoid local blisters. The duration of treatment should be dependent on the herb potency and skin tolerability. There is a little warm feeling, burning sensation, or distending pain in the vicinity of acupoints during the therapy [7]. The paste should be removed immediately when patients cannot stand pain. Moreover, try not to scratch the skin of the selected acupoints. The skin could have left scars if pasted too long (Figure 4).

**Figure 5:**
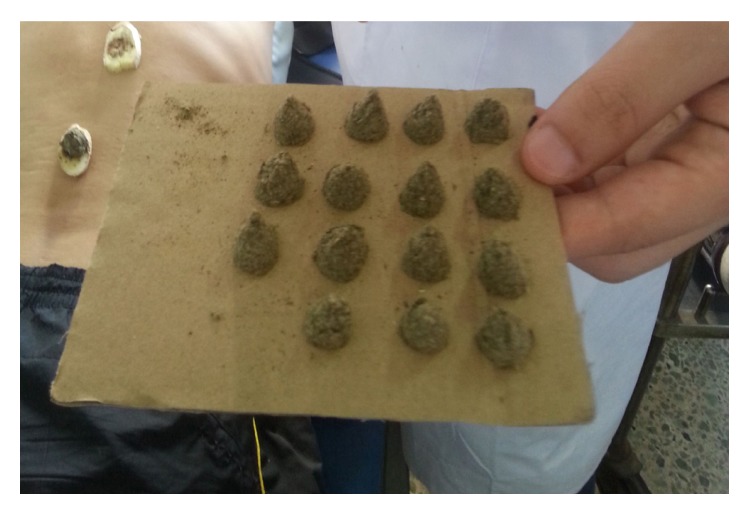
Moxa cones for San-Fu-Jiu.

**Figure 6:**
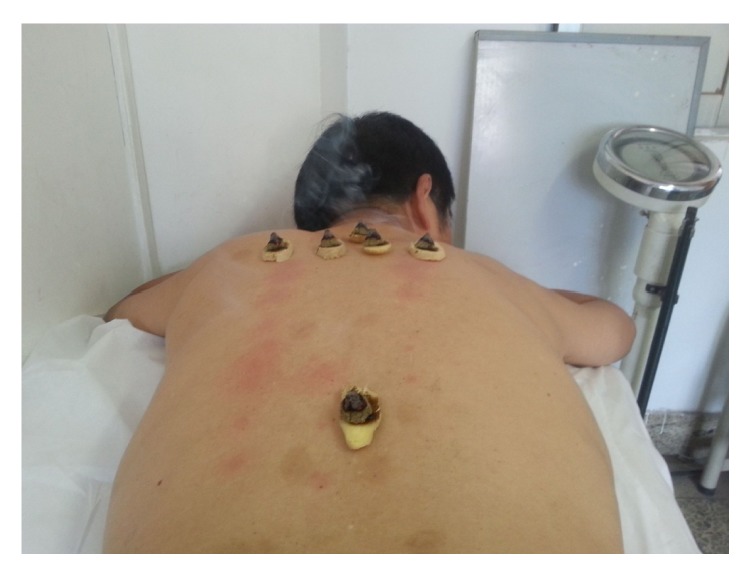
San-Fu-Jiu was being applied.

San-Fu-Jiu is another important therapy on the dog days. Moxa wool and fresh ginger are the main materials of San-Fu-Jiu. Firstly, fresh ginger is cut into about 1.5–2.5 cm in diameter, about 0.2–0.3 cm thick slices. Secondly, ginger slices are pierced by acupuncture needle to form small holes to facilitate heat transfer. Lastly, moxa wool is molded into moxa cones. A moxa cone of half an olive size with a diameter of 1–1.5 cm, weighing about 2 g, is placed on the ginger slice which is put on the selected acupoint and burned for moxibustion stimulation (Figure 5). Five to seven consecutive moxa cones are needed to be burned on every acupuncture point. Generally, the treatment lasts half an hour each time. Warm feeling, skin redness, and local blisters are normal phenomena (Figure 6) [7].

AR, a common chronic respiratory disorder, is characterized by sneezing, rhinorrhea, nasal congestion, and nasal pruritus [8]. Although it is not life-threatening, symptoms are bothersome and unbearable. In addition, it has a significant impact on work and quality of life, imposing a significant burden on both the families and society [9–12]. The antihistamine drugs or glucocorticoid was the common agents for patients of AR [13]. The short-term effect was satisfied. However, the long-term effect was unsatisfactory and recurrence rate was high [13]. Furthermore, those drugs may lead to medicamentous rhinitis over a long period of time. The studies showed that San-Fu-Tie and San-Fu-Jiu could reduce the rate of recurrence compared with western medicine [2]. San-Fu-Tie is applied onto the acupoints which are associated with AR. Meanwhile, San-Fu-Jiu could be applied in AR patients. With the patient in the prone position, some acupuncture points on the back are chosen to put ginger slices for moxibustion therapy.

Asthma is another chronic respiratory disorder in the world. It approximately affects 300 million people worldwide and will influence more in the next decades [14]. Being an extensive global health issue, uncontrolled asthma is associated with work productivity loss and poor quality of life. Moreover, a serious economic burden of asthma soars up in recent years [15]. Despite advances in the understanding and management of asthma, many patients were not available to antiallergic agents due to adverse effects [16]. As a complementary therapy, San-Fu-Tie or San-Fu-Jiu could strengthen physique with the purpose of preventing or reducing asthma attacks and had few side effects [17].

Despite the favorable therapeutic effect, patients with damaged skin or skin allergies should be treated with caution. Pregnant women, active pulmonary tuberculosis, and acute febrile patients are banned from acupuncture point application therapies. Patients had better not take spicy or irritant foods, cold drinks, and alcohol during treatment [7]. In addition, try to keep body warm and avoid catching cold on dog days.

While the effects of acupuncture point application therapies have been frequently reported in treating AR and asthma, there exist several issues. In this regard, the processes of San-Fu-Tie and San-Fu-Jiu had not been elucidated in detail. The majority of the clinical information derives from uncontrolled clinical trials or from retrospective reports, and few multicenter clinical trials have been conducted to confirm the effects of acupuncture point application therapies in the treatment of AR and asthma. In addition, the scientific evidence validating that acupuncture point application therapies are as effective as other conventional treatments in treating AR and asthma remains to be further validated. Given these issues, it is essential to assess the pertinent studies to systematically review the potential effects and safety of acupuncture point application therapies in the treatment of AR and asthma.

## 2. Materials and Methods

To ensure the accuracy of the systematic review, the results were designed and reported by employing a checklist of items that was as consistent as possible with the Preferred Reporting Items for Systematic Reviews and Meta-Analyses statement [18].

### 2.1. Search Strategy

We conducted a systematic search of the following databases to identify trials: PubMed, the Cochrane Library, and Clinical Trials.gov. In addition, the literatures were also collected from the Chinese databases: the CNKI Database, CBM Database, Wanfang Database, and Chinese Clinical Trial Register. All of the databases were searched from their available dates of inception to the latest issue (May 2015). For the English databases, free text terms were used, such as “Sanfutie,” “Sanfujiu,” or “Acupuncture point application therapies” and “Allergic rhinitis” or “Asthma.” For the Chinese databases, we used free text terms, such as “Sanfutie,” “Sanfujiu,” “Dong bing xia zhi,” or “Xue wei fu tie” (which are all alternative names for San-Fu-Tie or San-Fu-Jiu in Chinese) and “Guo min xing bi yan” or “Xiao chuan” (which means allergic rhinitis and asthma in Chinese, resp.).

### 2.2. Inclusion and Exclusion Criteria

The focus of the review was on studies of acupuncture point application therapies regardless of gender and publication status. All studies were required to fulfill the following inclusion criteria: (1) regardless of blinding or language, randomized controlled trials (RCTs) were included; (2) for the types of interventions, treatment with San-Fu-Tie or San-Fu-Jiu alone in RCTs was considered; (3) acupuncture points were the same every time during dog days; (4) the subjects only received treatment three times a year (treatment could be four times when dog days are four ten-day periods in some years).

Case reports, reviews, retrospective studies, open-label extension study, and studies without a control group were excluded.

### 2.3. Data Collection and Management

The search strategy, data collection, and management were executed by two independent reviewers and when divergences existed, a third reviewer was encouraged to achieve consensus. In the study published by Lin et al. [19], the subjects of three TCM syndrome patterns were pooled. For the trials that applied a three-armed group design [17, 20], only two groups were extracted while blank group was excluded. Information on population, interventions (including medicine, acupuncture points, and duration), outcomes, and adverse events was indicated in the tables (Tables 1 and 2). We adopted the validated Jadad instrument to evaluate the included studies' methodological quality [21]. The items of Jadad score are as follows: randomization (0–2 points); double-blinding (0–2 points); and description of withdrawals and dropouts (0-1 point). Allocation concealment (0–2 points) referred to the criterion of Schulz et al. [22]. Studies with Jadad scores of more than 3 were regarded as being of high quality.

## 3. Results

### 3.1. Study Selection

**Figure 7:**
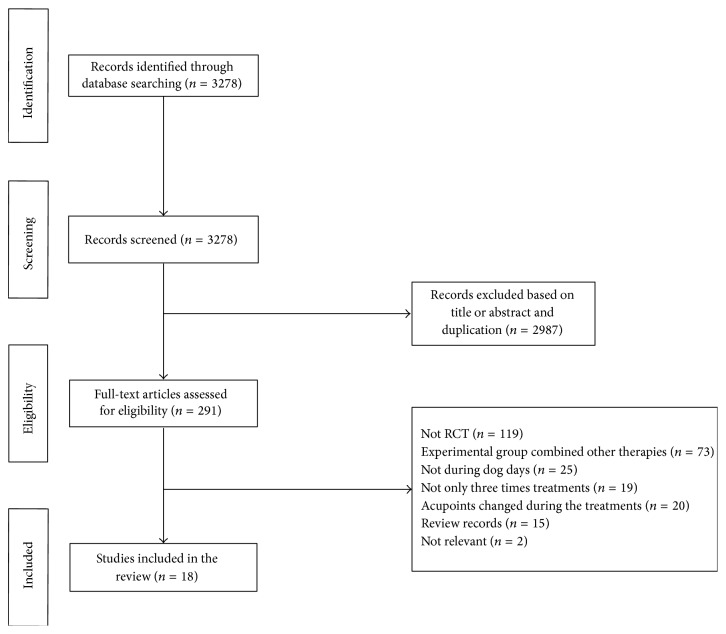
Process of searching for and screening studies.

The process of study selection was shown in Figure 7. According to the selection criteria defined in Materials and Methods, eighteen RCTs [17, 19, 20, 23–37] involving 1,785 subjects were included. Three of them [19, 23, 24] were about AR and acupuncture point application therapies. Together, those studies included a total of 333 participants. Fifteen RCTs [17, 20, 25–37] involving 1,452 participants were included with acupuncture point application therapies as intervention to treat asthma.

### 3.2. Study Descriptions

The included studies were published as full texts between 2008 and 2014. All of the RCTs originated in China and were performed as single-center trials, while three studies were in English and excluded as one [1] was a review article and two [3, 5] were not controlled trials.

### 3.3. Interventions and Controls

Four studies [26, 29, 30, 36] compared San-Fu-Tie with a placebo. Eleven studies [17, 19, 20, 23–25, 27, 28, 31, 35, 37] randomized the participants to receive San-Fu-Tie alone versus a control of western medicine. Three trials [32–34] compared San-Fu-Tie with a control of Chinese herbal medicine or acupuncture. All included studies were about San-Fu-Tie. As showed in Table 1, Feishu (BL13) was frequently applied to treat AR. As indicated in Table 2, the acupuncture points frequently selected to treat asthma were Feishu (BL13), Dazhui (GV14), Dingchuan (EX-B1), Shenshu (BL23), and Pishu (BL20).

### 3.4. Outcomes and Adverse Effects

The majority of the outcomes were efficacy evaluation and symptom rating scale. Twelve studies [19, 23–25, 28, 31–37] did not mention adverse effects. Four studies [17, 26, 29, 30] had no adverse effects. Two studies [20, 27] reported mild skin allergies or local swelling and blisters.

### 3.5. Quality of the Included Studies

Compared with the six trials [19, 26, 27, 29, 30, 36] that were of high quality, most of the included trials were of low quality (Jadad score < 3) because of unclear randomization, deficient allocation concealment, inadequate blinding, and undescribed withdrawals and dropouts.

### 3.6. Effects of Interventions

All the included studies indicated that acupuncture point application therapies were more effective and superior than various control groups regarding clinical symptoms or objective outcomes. They displayed significant differences between experimental group and control group.

## 4. Discussion

Acting on the recommendation of China Association for Acupuncture and Moxibustion and China Academy of TCM, acupuncture point application therapies are suitable for chronic and refractory respiratory diseases including AR and asthma [7]. Meanwhile, San-Fu-Tie and San-Fu-Jiu belong to the transdermal drug delivery. The transdermal drug delivery can be absorbed into circulatory system from the local capillary [38]. Compared with oral route, the route of drug administration offers pharmacological advantages in decreasing the irritation of digestive tract and liver [16]. As shown in the results, the minority of studies reported mild adverse effects. Consequently, San-Fu-Tie and San-Fu-Jiu could improve patients' acceptability and compliance.

According to the TCM, Yin and Yang are ubiquitous in the body and the environment. When deficiency of Yang fails to control Yin, some diseases always recur in winter, such as AR and asthma. In TCM theory, dog days are the hottest periods which are characterized by abundant Yang, skin and muscles being loose. Therefore, it is easy for body to absorb drugs [3, 5]. Applying herbal medicine, pungent in the taste and hot or warm in the nature onto the special acupoints, could help body absorb Yang from the environment, strengthen Yang inside the body, and maintain the functional status of Yin and Yang [1, 39]. Consequently, the body's Yang could be improved to defense against the diseases which occur in the cold days and to rebalance the Yin and Yang. With the assistance of external environment, herbs and acupoints stimulation play a paramount role at a specific time.

San-Fu-Tie and San-Fu-Jiu are two different patterns of acupuncture point application therapies during dog days. San-Fu-Tie is more simple and convenient to operate than San-Fu-Jiu. Therefore, the former is more widespread used for both clinical practice and clinical research. As shown in Tables 1 and 2, all included studies were concerning the application of San-Fu-Tie. Two studies applying San-Fu-Jiu were excluded as one was applied for cervical spondylosis [40] and the other one was a review article [41].

According to the theory of treating different diseases with the same method in TCM, these acupuncture points applied for asthma were similar with those for AR. In fact, the relevant acupuncture points are not limited to those shown in the tables. Yingxiang (LI20), located beside the midpoint of the nasal ala and among the nasolabial groove, was an important acupoint which is able to rapidly relieve the symptoms of AR. However, the majority of studies did not select Yingxiang (LI20). They may take the aesthetic judgments into account.

The data of the included studies was not pooled owing to their different interventions (including acupuncture points and Chinese herb medicine), comparisons, and outcomes. Acupuncture point application therapies could be superior to other therapies in the treatment of AR and asthma based on all included studies.

However, there are still some limits. Firstly, all the participants were recruited from Chinese populations, which implied a high risk of selection bias. Secondly, the majority of the studies were of poor quality. Only four studies [26, 29, 30, 36] applied an adequate blinding and three studies [19, 27, 36] performed allocation concealment. Therefore, potential bias, such as that in the selection of patients, the administration of interventions, and assessment of outcomes, could have resulted in the overestimation of the therapeutic efficacy of San-Fu-Tie. Thirdly, the herbal formula, acupuncture points, and outcomes are too incongruous to pool them. Therefore, it is necessary for all conclusions to be carefully explained.

## 5. Conclusion

In summary, the narrative review elaborates the operating process and contraindications of San-Fu-Tie and San-Fu-Jiu in detail. This systematic review suggests that San-Fu-Tie and San-Fu-Jiu have been widely employed in the treatment of AR and asthma characterized by favorable treatment effect, convenient operation, and few side effects. Consequently, it is worth spreading and utilizing in clinic. However, the outcomes of the included studies were not pooled due to their inconsistency. Given the low methodological quality of the randomized trials, large and well-designed RCTs are needed to confirm our conclusions.

## Figures and Tables

**Table 1:** AR and acupuncture point application therapies.

Author	Number and range of age		Intervention (herbal medicine and acupoints) and duration		Outcomes	Adverse events (experimental)	Jadad score
	Experimental	Control	Experimental	Control			
Zhu 2010 [23]	47/5–55	44/8–50	San-Fu-Tie, for one year	Cetirizine and beclometasone dipropionate aqueous nasal spray, for ten consecutive days	Efficacy evaluation	Not mentioned	2
			Bai Jie Zi, Xi Xin, Gan Sui, Yan Hu Suo, Ban Xia				
			GV14, BL20, CV17, BL12, BL13, BL23				

Chen and Gu 2011 [24]	40/4–7	34/4–7	San-Fu-Tie, for two-three consecutive years	Oxymetazoline hydrochloride spray and dexamethasone, withdrawal when symptomatic relief occurs	Efficacy evaluation	Not mentioned	2
			Bai Jie Zi, Xi Xin, Ma Huang, Xin Yi, Sheng Jiang				
			GV14, BL13, BL43, PC6				

Lin et al. 2014 [19]	84/5–82	84/5–82	San-Fu-Tie, four times a year (dog days are four ten-day periods in 2013), for one year	Beclometasone dipropionate aqueous nasal spray, for four weeks	Efficacy evaluation, symptom rating scale	Not mentioned	4
			Bai Jie Zi, Xi Xin, Ma Huang, Gan Sui, Yan Hu Suo, Ding Xiang, Sheng Jiang				
			BL13, BL20, GV14, BL23, BL12, LI4, EX-B1, ST36, EX-HN15				

Bai Jie Zi, *Semen Sinapis Albae*; Xi Xin, *Herba Asari*; Gan Sui, *Radix Kansui*; Yan Hu Suo, *Rhizoma Corydalis*; Sheng Jiang, *Rhizome Zingiberis Recens*; Ban Xia, *Rhizoma Pinelliae*; Ma Huang, *Herba Ephedrae*; Xin Yi, *Flos Magnoliae*; Ding Xiang, *Flos Caryophylli*.

BL13, Feishu; BL43, Gaohuang; EX-HN15, Bailao; CV17, Danzhong; EX-B1, Dingchuan; GV14, Dazhui; BL20, Pishu; BL12, Fengmen; BL23, Shenshu; PC6, Neiguan; LI4, Hegu; ST36, Zusanli.

AR, allergic rhinitis; cAMP, cyclic Adenosine Monophosphate; cGMP, cyclic Guanosine Monophosphate.

**Table 2:** Asthma and acupuncture point application therapies.

Author	Number and range of age		Intervention (herbal medicine and acupoints) and duration		Outcomes	Adverse events (experimental)	Jadad score
	Experimental	Control	Experimental	Control			
Cai 2008 [17]	40/2–12	40/2–12	San-Fu-Tie, for one year Bai Jie Zi, Ma Huang, Tan Xiang EX-B1, BL20, BL23, GV14, BL13	BCG polysaccharide and nucleic acid injection, twice a week, for three months	Symptom rating scale, IL-4, IFN-*γ*	No	2

Mou et al. 2009 [25]	91/18–65	55/18–65	San-Fu-Tie, for three consecutive years Bai Jie Zi, Xi Xin, Gan Sui, Yan Hu Suo, Sheng Jiang, Rou Gui, Huang Qi, Dang Shen, Shan Yao, Gan Cao, Bing Pian BL13, EX-B1, CV17, CV22, LU1	Oral preparation of traditional Chinese medicine, three months in one year, for three consecutive years	Efficacy evaluation, symptom rating scale	Not mentioned	2

Cai et al. 2010 [26]	60/3–14	60/3–14	San-Fu-Tie, for one year Bai Jie Zi, Ma Huang, Tan Xiang EX-B1, BL20, BL23, GV14, BL13	PBO, three times a year, for one year EX-B1, BL20, BL23, GV14, BL13	Symptom rating scale, safety assessment	No	4

Li and Yuan 2010 [27]	30/3–8.2	30/3.2–8.5	San-Fu-Tie, for one year Bai Jie Zi, Ma Huang, Xi Xin, Sheng Jiang, Jiang Can, Feng Mi BL13, CV17, CV22, BL17	BCG Polysaccharide and nucleic acid injection, once every other day, for one month	Efficacy evaluation, the average number of cold, IgA, IgG, IgM	One patient had mild skin allergies	4

Zhang 2010 [28]	30/18–70	30/18–70	San-Fu-Tie, for three consecutive years Bai Jie Zi, Xi Xin, Yan Hu Suo BL13, EX-B1, BL20, CV4	Ketotifen, July to September each year, for three consecutive years	Efficacy evaluation	Not mentioned	2

Tao 2012 [29]	60/3–14	60/3–14	San-Fu-Tie, for one year Bai Jie Zi, Ma Huang, Tan Xiang BL23, GV14, BL13, EX-B1, BL20	PBO, three times a year, for one year BL23, GV14, BL13, EX-B1, BL20	Symptom rating scale	No	4

Cui 2012 [20]	28/2–14	28/2–14	San-Fu-Tie, for one year Bai Jie Zi, Xi Xin, Gan Sui, Wu Zhu Yu, Sheng Jiang, Su Zi, She Xiang BL13, BL12, BL43	Pulmicort, for one year	Symptom rating scale, IgA, IgE, IgG, IgM	Two patients had local swelling and blisters	2

Li 2012 [30]	60/3–14	60/3–14	San-Fu-Tie, for one year Bai Jie Zi, Ma Huang, Tan Xiang BL23, GV14, BL13, EX-B1, BL20	PBO, three times a year, for one year BL23, GV14, BL13, EX-B1, BL20	Symptom rating scale, safety assessment	No	4

Zhu and Chen 2012 [31]	36/18–65	36/19–63	San-Fu-Tie, for one year Bai Jie Zi, Xi Xin, Gan Sui, Yan Hu Suo, Sheng Jiang, Ma Huang, Ban Xia BL13, CV17, CV22, LU1, GV14, BL17, EX-HN15, BL12	Salbutamol, withdrawal when symptomatic relief occurs	Efficacy evaluation	Not mentioned	1

You et al. 2012 [32]	50/3–80	30/5–81	San-Fu-Tie, for three consecutive years Bai Jie Zi, Xi Xin, Gan Sui, Sheng Jiang, Huang Qi BL12, BL13, EX-B1, BL20, CV4, BL23	Yupingfeng granules, thirty days during dog days every year, for three consecutive years	Efficacy evaluation	Not mentioned	2

Chen et al. 2013 [33]	56/3–14	30/2.8–14	San-Fu-Tie, for one year Bai Jie Zi, Xi Xin, Gan Sui, Yan Hu Suo, Sheng Jiang, Su Zi BL13, BL15, BL17	Yupingfeng granules, for thirty days during dog days, for one year	Efficacy evaluation, IgA, IgE, IgG, IgM	Not mentioned	2

Z. P. Zhang and H. Y. Zhang 2013 [34]	60/18–65	60/18–65	San-Fu-Tie, for three consecutive years Bai Jie Zi, Xi Xin, Gan Sui, Wu Zhu Yu BL13, BL20, GV14, BL23, CV22, BL12	Acupuncture therapy, thirty days during dog days every year, for three consecutive years BL13, BL20, GV14, BL23, CV22, BL12	Efficacy evaluation	Not mentioned	2

Hu 2014 [35]	50/5–12	50/5–12	San-Fu-Tie, for one year Bai Jie Zi, Xi Xin, Gan Sui, Yan Hu Suo BL13, BL20, GV14, BL11	Salmeterol xinafoate and fluticasone propionate powder for inhalation, for one year	Attack frequency, FEV_1_, FVC, PEFR	Not mentioned	2

Shi and Zhao 2014 [36]	46/3–14	46/2.8–14	San-Fu-Tie, for one year Bai Jie Zi, Xi Xin, Gan Sui, Yan Hu Suo, Sheng Jiang, Fang Feng, Bing Pian BL13, BL15, BL17	PBO, three times a year, for one year BL13, BL15, BL17	Efficacy evaluation, FEV_1_, PEF, FVC, FEV_1_/FVC, EOS	Not mentioned	5

Chen 2014 [37]	70/4–12	70/4–12	San-Fu-Tie, for one year Bai Jie Zi, Ma Huang, Tan Xiang BL13, BL20, GV14, BL23, EX-B1	BCG polysaccharide and nucleic acid injection, twice a week, for three months	Symptom rating scale, IL-4, IFN-*γ*	Not mentioned	2

Bai Jie Zi, *Semen Sinapis Albae*; Xi Xin, *Herba Asari*; Gan Sui, *Radix Kansui*; Yan Hu Suo, *Rhizoma Corydalis*; Sheng Jiang, *Rhizome Zingiberis Recens*; Ban Xia, *Rhizoma Pinelliae*; Ma Huang, *Herba Ephedrae*; Tan Xiang, *Lignum Santali Albi*; Rou Gui, *Cortex Cinnamomi*; Huang Qi, *Radix Astragali*; Dang Shen, *Radix Codonopsis*; Shan Yao, *Rhizoma Dioscoreae*; Gan Cao, *Radix Glycyrrhizae*; Bing Pian, *Borneolum Syntheticum*; Jiang Can, *Bombyx Batryticatus*; Feng Mi, *Mel*; Wu Zhu Yu, *Fructus Evodiae*; Su Zi, *Fructus Perillae*; She Xiang, *Moschus*; Fang Feng, *Radix Saposhnikoviae*.

BCG, Bacillus Calmette-Guérin; IL-4, interleukin-4; IFN-*γ*, interferon-*γ*; PBO, placebo; FEV_1_, Forced Expiratory Volume in One Second; FVC, Forced Vital Capacity; PEFR, Peak Expiratory Flow Rate; PEF, Peak Expiratory Flow; EOS, eosinophils; WBC, white blood cell; NEUT, neutrophil; LYM, lymphocyte; MONO, monocyte; RBC, red blood cell; HGB, hemoglobin; PLT, blood platelet.

BL13, Feishu; BL43, Gaohuang; EX-HN15, Bailao; CV17, Danzhong; EX-B1, Dingchuan; GV14, Dazhui; BL20, Pishu; BL12, Fengmen; BL23, Shenshu; PC6, Neiguan; LI4, Hegu; ST36, Zusanli; CV22, Tiantu; LU1, Zhongfu; BL17, Geshu; CV4, Guanyuan; BL15, Xinshu; BL11, Dazhu.
